# Supplementary material for: D-Loop Mutation G42A/G46A Decreases Actin Dynamics
Source: Biomolecules. 2020 May 8;10(5):736. doi: 10.3390/biom10050736 (PMC7277580; doi:10.3390/biom10050736)
Supplement: Supplementary file 1 [file biomolecules-10-00736-s001.zip › supplementary biomolecules-777354.docx]

**Supplementary Materials**

**Movie S1.** Time-lapse movie (1140 s total) of 0.3 μM actin filament (including 20% Alexa Fluor 488-labeled skeletal muscle actin and 80% non-labeled wild-type β-actin) polymerization and depolymerization (related to Fig. 4A). Actin filaments were tethered on a glass surface with 50 nM NEM-myosin. Depolymerization was induced by loading TIRF buffer (10 mM imidazole, 50 mM KCl, 1 mM MgCl_2_, 1 mM EGTA, 100 mM DTT, 0.2 mM ATP, 0.02 mM CaCl_2_, pH 7.0) to remove any free actin monomer (indicated by "Buffer").

**Movie S2.** Time-lapse movie (1140 s total) of 0.3 μM actin filament (including 20% Alexa Fluor 488-labeled skeletal muscle actin and 80% non-labeled mutant β-actin) polymerization and depolymerization (related to Fig. 4B). Actin filaments were tethered on a glass surface with 50 nM NEM-myosin. Depolymerization was induced by loading TIRF buffer (10 mM imidazole, 50 mM KCl, 1 mM MgCl_2_, 1 mM EGTA, 100 mM DTT, 0.2 mM ATP, 0.02 mM CaCl_2_, pH 7.0) to remove any free actin monomer (indicated by "Buffer").
